# Presence and absence of light-independent chlorophyll biosynthesis among *Chlamydomonas* green algae in an ice-covered Antarctic lake

**DOI:** 10.1080/19420889.2019.1676611

**Published:** 2019-10-13

**Authors:** David Roy Smith, Marina Cvetkovska, Norman P. A. Hüner, Rachael Morgan-Kiss

**Affiliations:** a Department of Biology, University of Western Ontario, London, Ontario, Canada; b Department of Biology, University of Ottawa, Ottawa, Ontario, Canada; c Department of Microbiology, Miami University, Oxford, OH, USA

**Keywords:** Chlorophyll, DPOR, ICE-MDV, photosynthesis, psychrophile, UWO241

## Abstract

The cold, permanently ice-covered waters of Lake Bonney, Antarctica, may seem like an uninviting place for an alga, but they are home to a diversity of photosynthetic life, including *Chlamydomonas* sp. UWO241, a psychrophile residing in the deep photic zone. Recently, we found that UWO241 has lost the genes responsible for light-independent chlorophyll biosynthesis, which is surprising given that this green alga comes from a light-limited environment and experiences extended periods of darkness during the Antarctic winter. Why discard such a process? We argued that it might be linked to the very high dissolved oxygen concentration of Lake Bonney at the depth at which UWO241 is found. Oxygen is the Achilles’ heel of the key enzyme involved in light-independent chlorophyll biosynthesis: DPOR. If this hypothesis is true, then other algae in Lake Bonney should also be susceptible to losing DPOR, such as *Chlamydomonas* sp. ICE-MDV, which predominantly resides in the chemocline, a depth with an even higher oxygen concentration than that where UWO241 exists. Here, we report that, contrary to our earlier prediction, ICE-MDV has maintained the genes encoding DPOR. We briefly discuss the implications of this finding in relation to the loss of light-independent chlorophyll synthesis in UWO241.

## Main document

No two lakes are the same. But Lake Bonney in the McMurdo Dry Valleys of Victoria Land, Antarctica, is perhaps one of the most unique and uninviting lakes on Earth. Its frigid waters (around 40 meters deep) are permanently enclosed by one storey of ice, resulting in a chemically stratified environment, largely cut off from the outside world [1]. Nevertheless, Lake Bonney is home to an array of microbial life [2], including eukaryotic algae [3–5]. Among the best-studied algae that brave the lake’s waters are *Chlamydomonas* sp. UWO241 [6,7] and *Chlamydomonas* sp. ICE-MDV [5,8]. Both are bona fide psychrophiles in that they can withstand intense cold but die at more moderate temperatures.

There are better places than an ice-covered Antarctic lake to perform photosynthesis. Within Lake Bonney, UWO241 and ICE-MDV must endure sustained cold (~5°C year-round), high salinity (up to 0.7 M NaCl in the deep layers), and perpetual low irradiance, not to mention 24-hours of darkness during peak austral winter [1]. Given these conditions, one might assume that being able to synthesize chlorophyll in the dark would be a major asset for an alga that calls Lake Bonney home. But recently, to our great surprise, we discovered that UWO241 has lost the ability to do just that, and we hypothesized that ICE-MDV has as well [9]. Here, we briefly update our findings, showing that our prediction for ICE-MDV was wrong, which may have implications for how we interpreted the loss of light-independent chlorophyll synthesis in UWO241.

Most eukaryotic algae contain two distinct nonhomologous enzymes for the penultimate step of chlorophyll *a* biosynthesis: light-dependent and light-independent protochlorophyllide oxidoreductase (LPOR and DPOR, respectively) [10,11]. LPOR, which is encoded by the nuclear gene *por*, is ubiquitous among photosynthetic eukaryotes [12] and is only active when its pigment substrate (protochlorophyllide) absorbs light [13]. Evidence suggests that LPOR is three to seven times more efficient when protochlorophyllide absorbs red light (647 nm) relative to blue light (407 nm) [14], which penetrates deeper into the water column. Conversely, DPOR, which is encoded by the chloroplast genes *chlB, chlL*, and *chlN*, can facilitate chlorophyll synthesis in the dark [11], but has been lost multiple times independently throughout eukaryotic evolution, most notably in all angiosperms [12]. DPOR is also dependent on iron for constructing iron-sulfur clusters [15], which is not true of the iron-moiety-lacking LPOR.

Chloroplast and nuclear genome sequencing demonstrated that UWO241 contains *por* but lacks *chlB, chlL*, and *chlN*, indicating it has discarded DPOR and is now entirely dependent on LPOR for making chlorophyll [9]. Why would any self-respecting photosynthesizer living in a light-limited environment dispose of DPOR? We reasoned that it might be linked to the very high dissolved oxygen concentration of Lake Bonney, which is >1,000 μM over the first fifteen meters [16] and remains high (~200% air saturation) at 17 m, where UWO241 is primarily located. DPOR, which is thought to have first evolved in anoxygenic photosynthetic bacteria, is oxygen sensitive [17,18], whereas LPOR, which first evolved in cyanobacteria [19], is not [20]. If the high oxygen content of Lake Bonney inhibited DPOR in UWO241 then presumably there would be no additional deleterious effects resulting from mutations knocking out the genes for DPOR, or so went our argument. But if this hypothesis is correct then the DPOR from other algae in Lake Bonney should also be inhibited by the elevated oxygen levels and susceptible to loss.

So, what of ICE-MDV? It can be found in the shallow zone of Lake Bonney (5 m) and is particularly dominant in the chemocline (15 m) where the dissolved oxygen concentration is even higher than in the deeper photic zone where UWO241 exists [16]. Hence, we predicted that, like UWO241, sequencing of its chloroplast genome would reveal the loss of the three genes encoding DPOR. Fortunately, we were able to reach out to some colleagues who have carried out extensive next-generation sequencing on ICE-MDV (Ion Torrent sequencing using Hi-Q chemistry and a P1 chip as well as Illumina paired-end sequencing on a NextSeq500) and searched these data for evidence of DPOR. (Please see [8] for details on culture conditions, DNA isolation, and sequencing and assembly methods.)

Alas, complete sequences of *chlB, chlL*, and *chlN* were easily located in the draft genome assembly of ICE-MDV, which comprised 331,087 contigs, averaging 812 nt. Note: the sequences were identified by blasting (BLASTN) the UWO241 *chlB, chlL*, and *chlN* genes against the ICE-MDV contigs. Adding to our disappointment, these genes were clearly chloroplast located and harbored no signs of deleterious or knockout mutations (please see GenBank accessions MN046391-MN046393). A nuclear-located gene for *por* was also found in the assembly. Thus, despite existing in an aquatic environment with a remarkably high dissolved oxygen concentration, ICE-MDV clearly retains functional genes for light-dependent and light-independent chlorophyll synthesis.

What does this mean for our earlier conjectures on the forces driving the loss of DPOR in UWO241? Surely, it weakens them. But it is worth highlighting an additional feature of the ICE-MDV data we collected. Specifically, the ICE-MDV *chlB, chlL*, and *chlN* genes share 100%, 99.8%, and 100% nucleotide sequence identity, respectively, with those from another Antarctic green alga: *Chlamydomonas* sp. ICE-L [21]. (Nucleotide alignments were performed with ClustalW implemented through Geneious v10.2.6, Biomatters Ltd., New Zealand, using default settings). This implies that ICE-MDV and ICE-L are very closely related to one another, which is intriguing because ICE-L was isolated >2500 kilometers from Lake Bonney near Zhongshan Station, Antarctica, from the underside of Antarctic sea ice (where the oxygen concentrations are not extremely high) [22]. UWO241, on the other hand, belongs to a different chlamydomonadalean clade (the Moewusinia) than ICE-MDV and ICE-L (the Monadinia), and appears to represent a distinct lineage within that clade [6]. Lastly, a recent paper reported that UWO241 and ICE-MDV exhibit additional physiological differences [23].

The close phylogenetic affiliation of ICE-MDV and ICE-L could be an indication that the former is part of population that extends beyond the bounds of Lake Bonney – potentially into an environment where oxygen concentrations are low enough to not inhibit DPOR. If such a scenario is true, then it might be preventing the loss of the genes encoding for light-independent protochlorophyllide oxidoreductase within ICE-MDV. Alternatively, the close phylogenetic relationship between ICE-MDV and ICE-L could suggest that ICE-MDV arrived in Lake Bonney more recently than UWO241 and has not yet had time to lose the *chlB, chlL*, and *chlN* genes. Moreover, we currently do not know if DPOR is active in the high-oxygen natural habitat of ICE-MDV and whether this alga has retained the ability to synthesize chlorophyll in the absence of light. Whatever the reasons for the presence of DPOR in ICE-MDV and its absence in UWO241, these data further emphasize just how unique UWO241 is relative to other green algae [7], both inside and outside of Lake Bonney.
